# Independent Control Over Size and Surface Density of Droplet Epitaxial Nanostructures Using Ultra-Low Arsenic Fluxes

**DOI:** 10.3390/nano11051184

**Published:** 2021-04-30

**Authors:** Sergey V. Balakirev, Natalia E. Chernenko, Mikhail M. Eremenko, Oleg A. Ageev, Maxim S. Solodovnik

**Affiliations:** Institute of Nanotechnologies, Electronics and Equipment Engineering, Southern Federal University, 2 Shevchenko St., 347922 Taganrog, Russia; nchernenko@sfedu.ru (N.E.C.); eryomenko@sfedu.ru (M.M.E.); ageevoa@inbox.ru (O.A.A.)

**Keywords:** droplet epitaxy, nanostructures, indium, gallium arsenide, arsenic flux

## Abstract

Modern and future nanoelectronic and nanophotonic applications require precise control of the size, shape and density of III-V quantum dots in order to predefine the characteristics of devices based on them. In this paper, we propose a new approach to control the size of nanostructures formed by droplet epitaxy. We reveal that it is possible to reduce the droplet volume independently of the growth temperature and deposition amount by exposing droplets to ultra-low group-V flux. We carry out a thorough study of the effect of arsenic pressure on the droplet characteristics and demonstrate that indium droplets with a large initial size (>100 nm) and a low surface density (<10^8^ cm^−2^) are able to shrink to dimensions appropriate for quantum dot applications. Small droplets are found to be unstable and difficult to control, while larger droplets are more resistive to arsenic flux and can be reduced to stable, small-sized nanostructures (~30 nm). We demonstrate the growth conditions under which droplets transform into dots, ring and holes and describe a mechanism of this transformation depending on the ultra-low arsenic flux. Thus, we observe phenomena which significantly expand the capabilities of droplet epitaxy.

## 1. Introduction

Semiconductor quantum dots (QDs) traditionally obtained by the Stranski–Krastanov growth mechanism have a large number of advantages over volume semiconductors or quantum two and one-dimensional nanostructures, such as narrow emission spectra, sharp density of states, broad excitation profiles, high extinction coefficient, etc. [1]. However, using the Stranski–Krastanov method, it is difficult to fabricate low-density arrays (~10^8^ cm^−2^ or less) of optically active QDs with dimensions close to 20 nm [2,3,4,5], which are very good candidates for use in single-photon emitters and sources of entangled photons [6,7]. Because of the large distance between QDs in a low-density array, it is possible to fabricate multiple separate devices using a single heterostructure divided into areas corresponding to the quantity of QDs. Then, the yield of areas containing a single QD is expected to be at a high level, especially in case of site-controlled growth on pre-patterned substrates.

QD arrays with an ultra-low surface density can be formed by the method of droplet epitaxy [8,9,10,11,12,13,14], which also has additional advantages over the Stranski–Krastanov growth method. Opportunities provided by droplet epitaxy include the possibility for QD growth without a wetting layer [10,15,16,17], the fabrication of nanostructures and complexes of nanostructures of various shapes [11,14,17,18,19,20,21] and QD growth in lattice-matched material systems, such as GaAs/AlGaAs, etc. [12,16,22,23]. The flexibility of droplet epitaxy makes it an advanced growth technique that is particularly attractive for use in the formation of single QDs [22,24,25].

It is commonly known that the higher the temperature used during growth by any of the techniques (Stranski–Krastanov or droplet epitaxy), the lower the surface density and the larger the average size of islands become [24,26,27,28,29]. In order to avoid the inter-dependence of the island size and surface density, various approaches have been applied, such as growth on patterned substrates [30,31], the use of subcritical deposition amounts [27,29,32,33], growth on metal-stabilized surfaces [34,35], etc. However, the simultaneous achievement of a low density and small size of nanostructures is still a relevant problem.

During droplet epitaxy, the exposure of droplets to group-V flux is normally conducted to turn metallic islands into III-V nanostructures, such as dots, rings, disks, etc. Any deviations from the dot shape are associated with the phenomenon of the diffusion decay of a droplet under group-V flux [36,37,38]. In order to suppress this effect to form a nanostructure with the shape of a dot, it is necessary to implement crystallization under a high group-V pressure (above 10^−5^–10^−4^ Pa) and at a low substrate temperature (below 250 °C) [26,31,39,40,41,42,43]. This enables the prevention of two-dimensional III–V growth during crystallization [26]. At the same time, the high intensity of atom diffusion from a metallic droplet is needed to form rings, disks and holes. These conditions are realized at high temperatures and low arsenic pressures [38,44,45]. Although several important models that are capable of predicting the final shape of III–V droplet epitaxial nanostructures depending on arsenic pressure have been carefully developed [45,46,47], they do not deal with very small arsenic fluxes that change the shape of metallic droplets without their significant crystallization.

In this work, we demonstrate a subtle method to use the arsenic pressure to alter droplet sizes while maintaining their surface density. Our technique implies the use of ultra-low arsenic fluxes (with values at least one order less than values used for the crystallization [26,39,40,41,42]) and allows the formation of low-density arrays of nanostructures with a small final size that are suitable for the further fabrication of optical QDs. We show that under certain conditions, such as surplus arsenic pressure, excess exposure time and a near-critical droplet size, it becomes difficult to control the droplet size because of a high probability of their complete decay. We propose a detailed mechanism of droplet behavior under the influence of ultra-low arsenic flux and demonstrate growth conditions under which the achievement of the best parameters of nanostructures is possible.

## 2. Materials and Methods

The samples were grown on epi-ready GaAs(001) substrates in a SemiTEq STE35 conventional molecular beam epitaxy system STE 35 (SemiTEq, Saint Petersburg, Russia). A valved cracker cell was used as the As source, and the As_4_ flux intensity was precisely controlled by varying the valve position. Native surface oxides were removed by heating the substrate up to 600 °C under an abundant As_4_ flux. Then, a GaAs buffer layer with a thickness of 400 nm was grown at a temperature of 580 °C and a growth rate of 1 monolayer (ML) per second. After the buffer layer growth, the substrate was cooled down to a deposition temperature of 300 °C with the valve fully closed. The choice of this deposition temperature was associated with the surface density of droplets observed after In deposition at this temperature (10^8^ cm^−2^ or less) [24,27,48]. At this density, the distance between droplets is more than 1 µm, which is convenient for the separation of quantum structures.

Then, the deposition of a given amount of indium expressed in equivalent InAs monolayers (from 1 to 3 ML) was carried out to form droplets. Immediately after their formation, droplets were exposed to an ultra-low arsenic flux of different values. The flux was estimated as an increase of the arsenic pressure measured by the vacuum gauge relative to the background pressure in the growth chamber right before the indium deposition. Ultra-small values of the arsenic flux implying a range from ~1 × 10^−7^ Pa to ~1 × 10^−6^ Pa make it possible to use an effect that is poorly observable when using large arsenic pressures. In the latter case, In droplets tend to become crystallized into InAs, changing their shape, or to etch the substrate, while slight fluxes of arsenic are capable of reducing a droplet in size without resulting in a substantial change in its shape. In this paper, we study the effect of the ultra-low arsenic flux on the parameters of nanostructures resting upon this fundamental difference.

It is well known that the elimination of arsenic pressure is needed to exclude the influence of arsenic on characteristics of metallic nanostructures [29]. However, it is quite difficult to determine an exact value of the arsenic pressure at which its effect can be neglected. Although the arsenic pressure continuously decreases after the valve is closed, the arsenic vapor is always present in the growth chamber and has a non-zero influence on the characteristics of metallic droplets. Nevertheless, a background pressure below 1 × 10^−7^ Pa is considered to be sufficient to implement the deposition of metallic droplets [29,34,35,36,37,38,39]. Thus, we used this value as a threshold for the indium deposition to ensure the absence of undesired arsenic pressure effects on the droplet characteristics of reference samples.

The reflection high-energy electron diffraction (RHEED) pattern prior to the indium deposition showed a clear (2 × 4) reconstruction of the GaAs(001) surface. The deposition of indium led to the disappearance of the crystalline surface structure, and the observation of a hazy spotty pattern by the RHEED system indicated the formation of a metallic phase on the surface [49,50]. The subsequent irradiation of indium droplets in the ultra-low arsenic flux did not lead to a substantial change in the RHEED pattern.

After the complete closure of the arsenic valve, the samples were held in the growth chamber during a time period that was equally predefined for each sample (5 min) while cooling down. Then, the substrates were transferred out of the growth chamber and sent to a scanning electron microscope (SEM) Nova NanoLab 600 (FEI Company, Eindhoven, The Netherlands) and atomic force microscope (AFM) NTEGRA (NT-MDT, Zelenograd, Russia) to measure the morphological characteristics of nanostructures.

## 3. Results and Discussion

**Figure 1:**
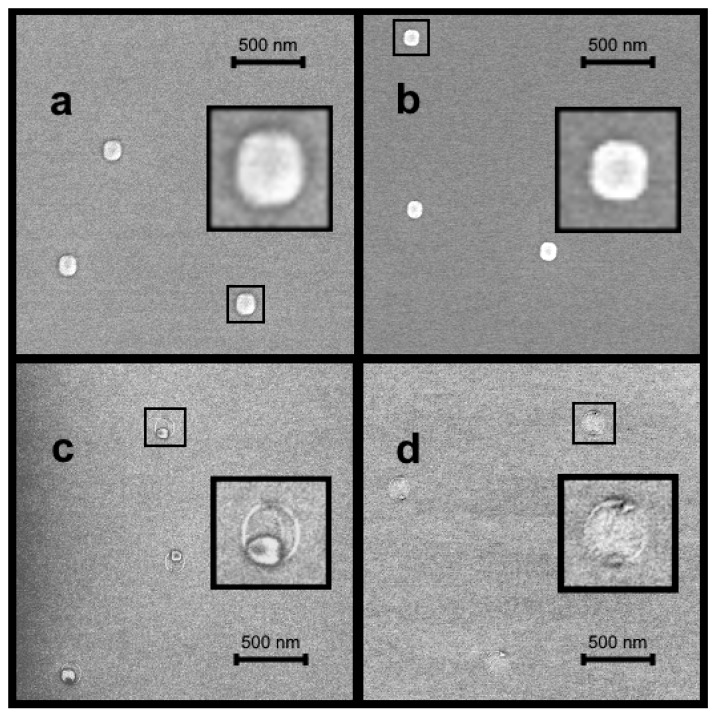
SEM images of nanostructures obtained after deposition of 3 ML of indium and further exposure to the ultra-low arsenic flux: (**a**) *P/P*_0_ = 1; (**b**) *P/P*_0_ = 4; (**c**) *P/P*_0_ = 6; (**d**) *P/P*_0_ = 8.1. The 250 × 250 nm^2^ insets demonstrate scaled-up nanostructures.

For the samples with 3 ML of deposited indium, a simple decrease of the average droplet size was observed with increasing arsenic pressure in a range of small values from *P/P*_0_ = 1 to *P/P*_0_ = 4 (Figure 1a,b). At larger arsenic fluxes, the ring formation occurred along the droplet perimeter, but the droplet still continued to decrease in size (Figure 1c,d). The fact that a droplet’s parameters change under the influence of temperature and arsenic flux is well known and mostly used for the purpose of nanoring [11,17,21,44] and nanohole [13,51,52] formation. This phenomenon is due to the following behavior of the growth system. The GaAs surface, which is initially arsenic-stabilized, becomes metal-stabilized after the In deposition. Then, droplets formed on the surface go into a stable equilibrium state in which the material balance is settled between the wetting layer (1 ML or more [24,27,29]) and droplets on the surface. Atoms do not migrate from the wetting layer to droplets because of their attraction by arsenic atoms in the substrate. At the same time, droplet atoms are in equilibrium with the wetting layer, and no concentration gradient appears. However, when the arsenic is supplied to the growth chamber, it covers the surface with an arsenic layer and partially penetrates into the droplets [53]. In this case, a concentration gradient arises between the arsenic-stabilized surface and metallic droplets, as a result of which a portion of the droplet atoms tend to occupy more energetically favorable positions on the surface arsenic atoms [54]. Thus, the atom leakage leads to a reduction of the droplet volume and the formation of a monolayer disk around the droplet [36,37].

**Figure 2:**
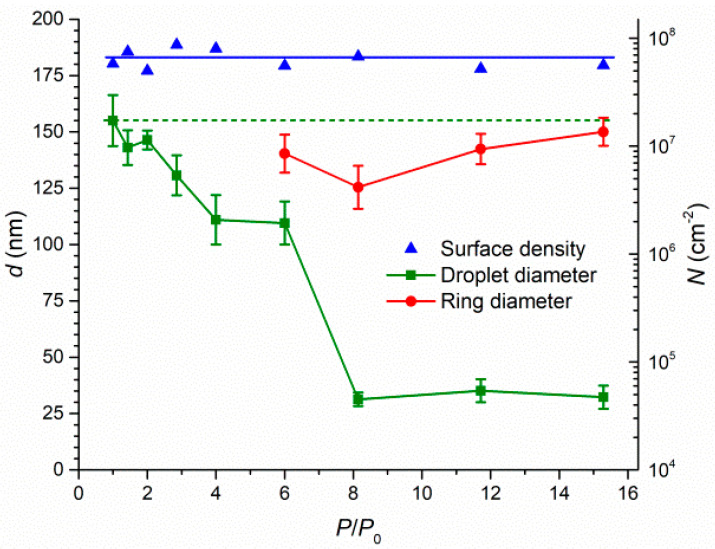
Pressure ratio dependences of the average size and surface density of droplets obtained after the deposition of 3 ML of indium and their further exposure to ultra-low arsenic flux. The caps represent standard deviations of the diameter of nanostructures.

Figure 2 demonstrates the arsenic flux dependences of the surface density and average diameter of droplets and rings after the deposition of 3 ML of indium. The droplets shrank over the entire range of the increase in the arsenic flux. At a sixfold increase of the arsenic pressure, the droplets transformed into a droplet–ring complex, meaning that the ring diameter was approximately equal to the initial droplet size. The ring formed at the interface of three phases (liquid droplet, vaporous arsenic and solid substrate) due to the increase in the arsenic concentration and thus the intensity of crystallization from In droplets to InAs.

One of the most important results we can observe in Figure 2 is the shrinkage of droplets without a ring formation around them. This phenomenon is realized below a certain threshold value of the arsenic pressure (*P/P*_0_ = 6 in Figure 2) above which the rings start to form. The boundary crystallization is a rapid process; therefore, there is a narrow range of arsenic fluxes at which the diffusion decay prevails over the crystallization. In this range, droplet shrinkage without ring formation is possible. However, even if a droplet simply reduces in size due to the diffusion decay, tracks of the crystallized boundary can appear within the initial droplet ring (shown in the inset in Figure 1c).

One can also observe in Figure 1c,d that decaying droplets stay on the droplet edges, not in the center of ring circles. Moreover, Figure 1d demonstrates that a droplet can form subdroplets in one direction ([011]) [36]. This phenomenon is attributed to the anisotropy of the surface diffusion of In adatoms under the influence of the arsenic flux on the GaAs(001) surface [36]. However, at the first stages of the diffusion decay, subdroplets do not form on both sides of the ring. Figure 1c clearly demonstrates that a shrunk droplet tends to remain on one edge of the ring. At the same time, droplets in Figure 1d split up into two halves. We suppose that this behavior is due to the secondary nucleation resulting from the significant transfer of In droplet material. Probably, the secondary nucleation is only possible in the case of the presence of the ring, which holds back In adatom flux and leads to its accumulation on the opposite side. Another possible scenario may be associated with the fact that, in some cases, a droplet may not decay immediately but remain resistant to the arsenic flux for some time. Upon reaching a certain critical value, the droplet abruptly falls apart into two small subdroplets located on opposite sides of the ring. This phenomenon requires further investigation to identify the most optimal conditions for the formation of nanodroplets.

An important observation that follows from Figure 2 is the saturation of the droplet size to a value of approximately 30 nm in the range of arsenic pressure ratios from 8 to 15.3. In this range, an increase in the arsenic flux does not lead to a noticeable decrease in the droplet size or to its decay. This behavior may be due to the fact that the droplet becomes crystallized earlier than the complete diffusion decay takes place. Although a thorough investigation of structural and compositional properties of such nanostructures is needed, the saturation phenomenon opens the way to a controlled reduction of the droplet size and good reproducibility of this process.

The mean standard deviation (indicated by caps in Figure 2) of the saturated diameter of droplets was found to be twice as much (4.4 nm) as for droplets exposed to lower arsenic fluxes (*P/P*_0_ = 6 and below; 8.8 nm on average). However, an increase in the arsenic flux does not lead to a monotonic change in the standard deviation of the droplet diameter until it reaches the saturation value (*P/P*_0_ = 8). The dispersion of ring diameters was observed to be at the level of the droplet size dispersion (7.7 nm) and tended to reduce with increasing arsenic flux. Discussing the results presented in Figure 2, it is also important to note that a small saturation size of nanostructures is achieved at a relatively high temperature without using a near-critical amount of deposited material, leading to the instability of formed droplets.

**Figure 3:**
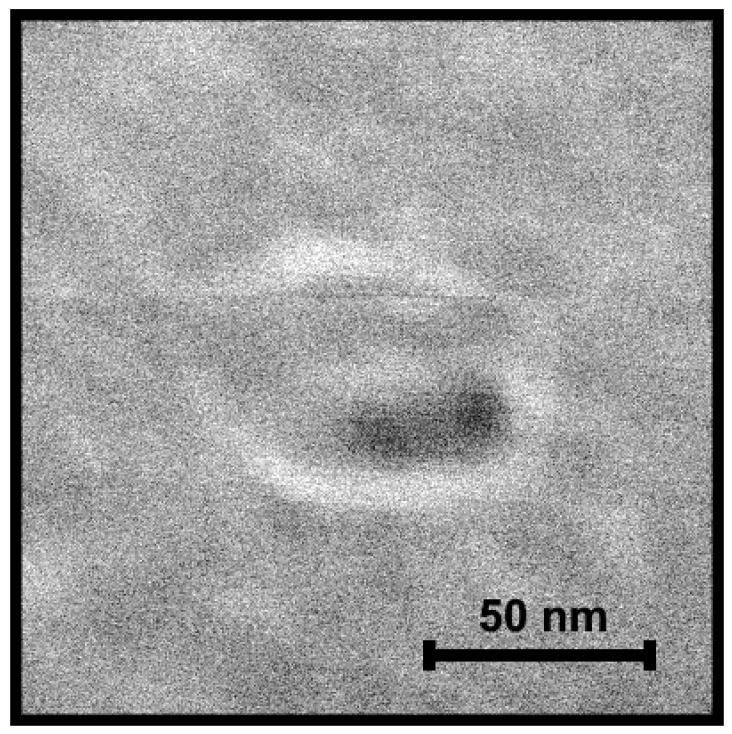
SEM image of a typical nanostructure after the exposure of In droplets obtained after the deposition of 1 ML of indium to the ultra-low arsenic flux.

Although the In/GaAs droplet system obtained after the deposition of 3 ML of indium allows a good understanding of what occurs on the surface under the influence of the ultra-low arsenic flux, 3 ML is a redundant amount of deposited material because it leads to the formation of droplets with a diameter of more than 100 nm in size. Using ultra-low arsenic fluxes, it is possible to decrease the droplet size to about 20 nm, which can be easily transformed into optically efficient InAs QDs [2,3,4,5]. However, excess droplet material is supposed to spread over the surface rather than evaporate in the chamber atmosphere [36,37]. In this case, the wetting layer may become thicker and have a negative influence on the heterostructure characteristics [16,55]. Thereby, we carried out studies on droplets obtained after the deposition of a minimal amount of material leading to droplet formation. According to our previous work [27], a critical deposition amount for the In/GaAs system at a temperature of 300 °C is 1 ML. After exposing critical droplets formed at this deposition amount to the ultra-low arsenic flux, we found out that these droplets were very unstable and completely decayed at an arsenic pressure ratio above 2. In order to retain the nanostructures, an extremely small arsenic pressure and exposure time must be provided. In this case, we were able to reveal an interesting effect: the formation of holes with surrounding rings (at a pressure ratio of 2.9, Figure 3) on the surface. Figure 3 shows that the holes formed on the edge of the ring as well as the droplets in a previous case. This confirms the logical conclusion that holes form at the droplet positions as a result of the substrate etching underneath [51,52]. It is important to note that the etching occurs mainly under the influence of high temperature. However, we observed that the hole formation occurred selectively even though the temperature was the same for all samples. Thus, holes did not necessarily form in the case of complete droplet decay.

Besides the temperature, the arsenic flux is one more important factor which has an influence on the droplet etching [56]. The exposure of metallic droplets to arsenic vapor gives rise to a number of microscopic events [56,57]. One of the most significant processes is caused by a change in the equilibrium concentration of As atoms in the metallic droplet as a result of the volume diffusion of As atoms into the droplet. An emerged displacement from the equilibrium state leads to the necessary compensation of deficient metallic atoms in order to restore the equilibrium composition. In this case, the nearest candidates for substitution are metallic atoms belonging to the substrate (Ga atoms in case of the In/GaAs system) [56]. Therefore, at a certain value of the arsenic flux, the equilibrium ratio of In and As atoms in the droplet is broken, and Ga atoms migrate from the substrate into the droplet volume with the further possibility of diffusion beyond its limits. In this case, a hole is formed at the place of the droplet (Figure 3).

**Figure 4:**
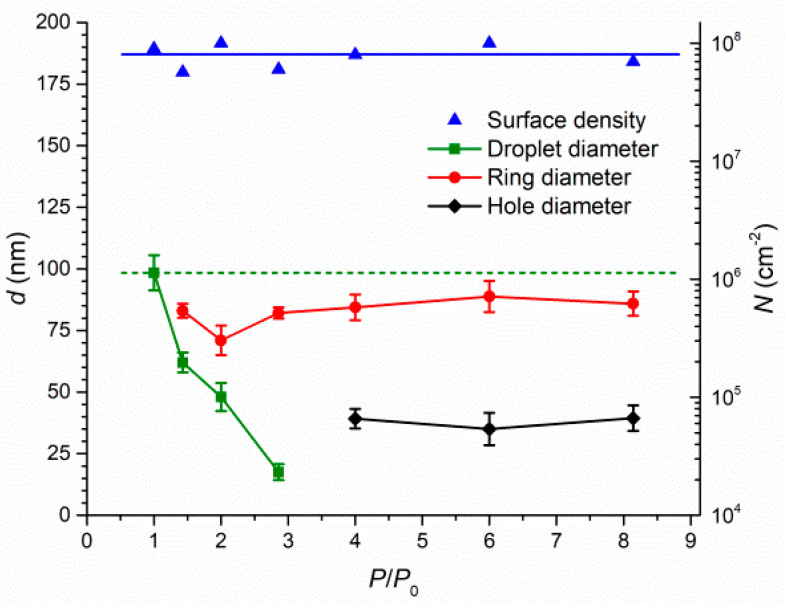
Pressure ratio dependences of the average size and surface density of droplets obtained after the deposition of 1.5 ML of indium and further exposure to ultra-low arsenic flux. The caps represent standard deviations of the diameter of nanostructures.

In order to carry out studies on more stable small droplets, we exposed droplets formed after the deposition of 1.5 ML of indium to the arsenic flux. The surface density was at the level of 7 × 10^−8^ cm^−2^ (Figure 4), as in the case of 3.0 ML (Figure 2). The arsenic flux dependences of the configuration and size of nanostructures were also in correlation with the 3 ML dependences. However, due to the fact that 1.5 ML droplets were still small and unstable, only one sample with droplets without rings around them was obtained. As well as in the case of the 3 ML deposition, the formation of rings around shrinking droplets was observed with increasing arsenic flux. However, the critical pressure value leading to droplet–ring formation was significantly shifted to the left, which was due to a decrease in the initial droplet volume. At arsenic pressure ratios above 4, holes formed in the place of droplets within the bounds of remaining rings. The mean standard deviations of the diameters of droplets, rings and holes were 5.0 nm, 4.6 nm and 5.3 nm, respectively, and these did not demonstrate an apparent upward or downward trend with increasing arsenic flux.

**Figure 5:**
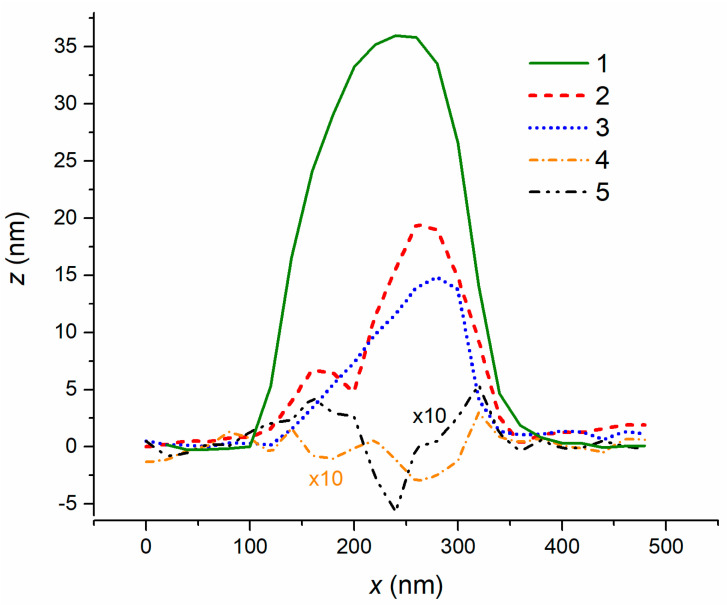
AFM profiles of nanostructures obtained after the deposition of 1.5 ML of indium and further exposure to the ultra-low arsenic flux: (**1**) *P/P*_0_ = 1; (**2**) *P/P*_0_ = 1.4; (**3**) *P/P*_0_ = 2; (**4**) *P/P*_0_ = 4; (**5**) *P/P*_0_ = 8.1.

AFM sections of 1.5 ML nanostructures (Figure 5) demonstrated that the nanostructure shrank both in diameter and height with increasing arsenic flux. Rings appeared in the place of the initial droplets while the droplet tended to remain on its edge. Nanostructures 4 and 5 combined to form a ring and a hole, which appeared in the place of a droplet. One can also observe that the ring height increased with the arsenic flux. This is associated with the fact that the crystallization processes on the three-phase boundary became more intensive, leading to an increase in the volume of InAs material. However, if the arsenic flux exceeded a certain threshold value, the crystallization could be ignored, giving way to the decay processes. Thus, there is a subtle boundary between the ring/hole formation and the complete diffusion decay of a droplet.

**Figure 6:**
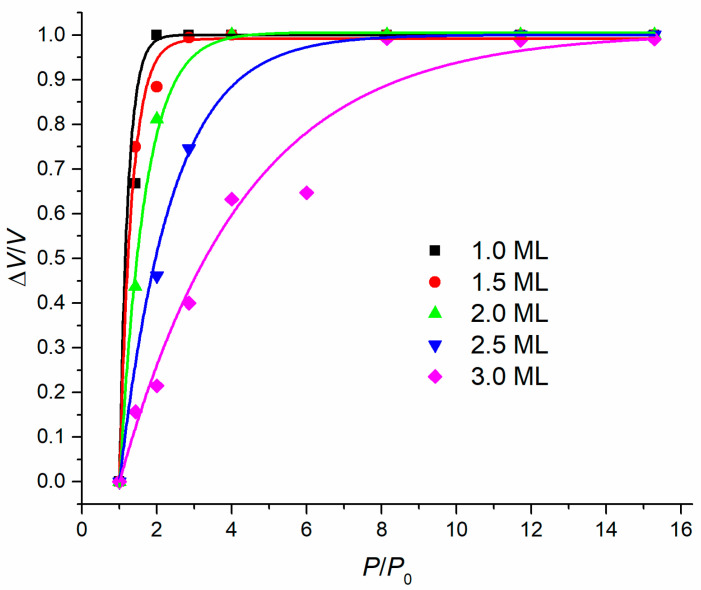
Pressure ratio dependences of a relative volume change of droplets obtained at various deposition amounts.

For the completeness of the study, we also exposed In droplets obtained at intermediate deposition amounts of 2.0 and 2.5 ML to the ultra-low arsenic flux. It is important to note that the surface density for all samples at each of the thicknesses varied around 7 × 10^−8^ cm^−2^, which indicates that the temperature fluctuations among the samples were quite small. Figure 6 presents the dependences of a relative change in the droplet volume on the arsenic flux for all deposition thicknesses under consideration.

The dependences reflect the rate of droplet diffusion decay under the influence of the arsenic pressure. While large droplets are able to withstand a large arsenic flux until they completely decay, small droplets are very susceptible, and it is very difficult to catch an intermediate position between entire droplets and monolayer disks or rings. At the same time, large droplets are followed by the deposition of excess indium material, which has a negative influence on the technology of nanostructure formation.

**Figure 7:**
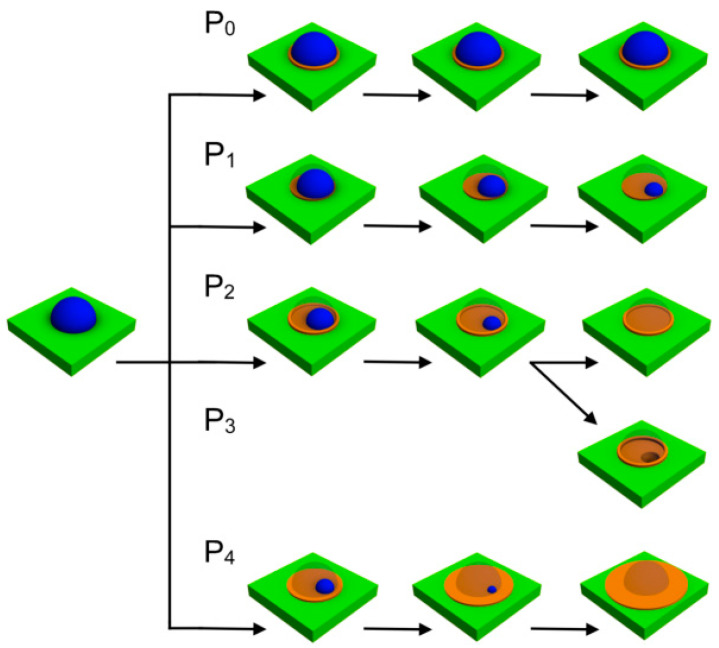
Schematic representation of the droplet diffusion decay under the ultra-low arsenic flux of different values where *P*_0_ < *P*_1_ < *P*_2_ < *P*_3_ < *P*_4_.

Summarizing all of the above-mentioned phenomena, we can demonstrate the patterns of behavior of In droplets under the influence of ultra-low arsenic flux. The first threshold value of the arsenic flux is when it is so small that droplets almost do not change in size; in this case, droplets are supposed to be crystallized only around the perimeter and come into equilibrium with the arsenic vapor (Figure 7, *P*_0_).

A slight increase in the arsenic flux leads to a slow diffusion decay of a droplet, which is realized through the droplet perimeter due to the concentration gradient between the arsenic-stabilized surface and the metal-rich droplet. As a result, the droplet decreases in size, maintaining its near-initial shape (Figure 7, *P*_1_). Although As atoms have an energetically favorable position at the droplet circle, the flux of As atoms is not enough to provide the formation of a distinct ring. However, it is sufficient to provide the arsenic stabilization of the surface after being covered by In atoms of the droplet. Thus, the diffusion decay is a more preferable process in this case.

A larger increase in the arsenic flux leads to the retention of As atoms at the droplet boundary and the formation of a stable InAs phase (Figure 7, *P*_2_). From this moment, the two processes start to compete for the material. On the one hand, In atoms leave the droplet, spreading over the surface or desorbing from it (if the temperature is high enough to activate the desorption). On the other hand, In atoms remain within the space of the initial droplet due to the crystallization into InAs. Taking into account the fact that the droplet does not cease to decay, we can conclude that a long exposure time eventually leads to the complete disappearance of the droplet. However, the InAs ring does not disappear once it has begun formation.

If the arsenic flux is even larger, a third process—namely the etching of the surface under the droplet—becomes significant (Figure 7, *P*_3_). In this case, the diffusion out of the droplet still occurs as well as the ring formation. However, the concentration of As atoms in the droplet becomes larger, resulting in a composition imbalance. Then, the droplet draws atoms out of the substrate and simultaneously decays, leaving behind a hole and a ring around the perimeter.

Thus, after a long exposure period, it is possible to observe a small droplet, a ring with a droplet, a ring with a hole or only a ring with a flat surface inside. However, there might be a situation in which the droplet decomposition rate is so large that neither crystallization nor etching can compete with it. This occurs when the arsenic flux exceeds a certain threshold value and simply spreads the droplets over the surface (Figure 7, *P*_4_).

## 4. Conclusions

The requirements for modern electronic and photonic devices motivate the search for non-trivial approaches to the synthesis of applied materials and structures. The precision control of single nano-objects is becoming a priority beyond the control of macroscopic parameters of nanostructure arrays. In this study, we developed a new approach to nanostructure modification which consists of the exposure of droplets that have formed after metal deposition to ultra-low group-V flux. This process results in a large number of advantages, including the independent control of the size and surface density of nanostructures, the reproducible formation of small-size droplets avoiding near-critical deposition amounts and the high-temperature droplet epitaxial synthesis of low-density QDs. This may be an efficient way to achieve the sufficient isolation of elements from each other, which is crucial for the fabrication of high-performance nanoelectronic and nanophotonic devices.

The shrinkage of droplets occurs due to the intense diffusion of atoms from the droplet under the influence of the arsenic flux. Despite the fact that this process is quite difficult to control, we revealed that a saturation of the droplet size is achieved in a certain range of fluxes, which is key to the reproducibility of this technological stage.

The stability of the process also depends significantly on the initial droplet volume. Small droplets obtained after a minimal amount of deposited material were found to be highly unstable. Therefore, we carried out a study of the effect of ultra-low arsenic flux on the characteristics of droplets formed by the deposition of various amounts of material. We demonstrated that a minimum value of the ultra-low arsenic flux and long exposure times should be used in order to obtain droplets of a small size to further crystallize them into optically efficient InAs QDs. Otherwise, the formation of rings and holes and their complexes is possible.

Although the revealed patterns relate to the InAs/GaAs material system, they can be successfully applied to other systems, such as GaAs/AlGaAs, GaSb/GaAs, InAs/InP, etc., which opens up great opportunities for the fabrication of highly efficient nanoelectronic and nanophotonic devices.
